# Salvia miltiorrhiza improves type 2 diabetes: A protocol for systematic review and meta-analysis

**DOI:** 10.1097/MD.0000000000023843

**Published:** 2021-02-12

**Authors:** Ying Guo, Jianfeng Sun, Renyan Zhang, Peng Yang, Sanyin Zhang, Zhipeng Wu

**Affiliations:** a School of Basic Medical Sciences, Chengdu University of Traditional Chinese Medicine; b Hospital of Chengdu University of Traditional Chinese Medicine; c Rehabitation Department of Chengdu Fifth People's hospital, Chengdu, Sichuan, PR China; d Innovative Institute of Chinese Medicine and Pharmacy of Chengdu University of Traditional Chinese Medicine.

**Keywords:** type 2 diabetes, Salvia miltiorrhiza, meta-analysis, protocol, randomized controlled trials, systematic review

## Abstract

**Background:**

Diabetes refers to any group of metabolic diseases characterized by high blood sugar and generally thought to be caused by insufficient production of insulin, impaired response to insulin. Globally, patients with type 2 diabetes account for more than 85% of the total diabetic patients, and due to factors, such as obesity, aging, environment and lifestyle, the incidence of diabetes is rising. Salvia miltiorrhiza (SM) is a medicine used to treat diabetes in China. In recent years, it has been reported that SM has the effect of improving type 2 diabetes. However, there is no systematic review of its efficacy and safety yet. Therefore, we propose a systematic review to evaluate the efficacy and safety of SM for T2D.

**Methods:**

Six databases will be searched: China National Knowledge Infrastructure (CNKI), China Biological Medicine (CBM), China Scientific Journals Database (CSJD), Wanfang database, PubMed, and EMBASE. The information is searched from January 2010 to July 2020. Languages are limited to English and Chinese. The primary outcomes include 2 hour plasma glucose, fasting plasma glucose, hemoglobin A1c, homeostasis model assessment of insulin resistance, and fasting plasma insulin. The secondary outcomes include clinical efficacy and adverse events.

**Results:**

This systematic review will evaluate the efficacy and safety of Salvia miltiorrhiza in the treatment of type 2 diabetes.

**Conclusion:**

This systematic review provides evidence as to whether Salvia miltiorrhiza is effective and safe for type 2 diabetes.

**Ethics:**

Ethical approval is not necessary as this protocol is only for systematic review and does not involve in privacy data or an animal experiment.

**Systematic review registration:**

INPLASY2020110046.

## 1 Introduction

Diabetes refers to any group of metabolic diseases characterized by high blood sugar and generally thought to be caused by insufficient production of insulin, impaired response to insulin. Globally, patients with type 2 diabetes account for more than 85% of the total diabetic patients,^[1]^ and due to factors, such as obesity, aging, environment and lifestyle, the incidence of diabetes is rising.^[2]^ Chronic hyperglycemia and insulin resistance can lead to chronic damage and dysfunction of various tissues, which seriously affects peoples quality of life. Long-term chronic damage and dysfunction are the main causes of death from diabetes.^[3]^ However, there are currently limited options of the drugs and treatment for diabetes, people are embarking on looking for alternative medicines from traditional herbal medicines.^[4]^

Salvia miltiorrhiza (SM) is a medicinal plant with a history of more than 2000 years of use in China. Traditional Chinese Medicine (TCM) uses SM to treat palpitation, stroke, perimenopausal syndrome, anemia and other diseases.^[5–7]^ Many clinical trials have found that SM has a significant improvement effect on type 2 diabetes.^[8–10]^

Unfortunately, there is currently no systematic review of the safety and effectiveness of SM in the treatment of T2D. Therefore, we propose a protocol for a systematic review to evaluate the effectiveness and safety of SM in the treatment of T2D patients, so as to provide a rigorous evaluation of the existing evidence.

## 2 Methods

### 2.1 Study registration

This protocol of systematic and meta-analysis review has been registered on Inplasy (https://inplasy.com/) with number INPLASY2020110046. Ethical approval is unnecessary because this study only involves the data of published previous studies.

### 2.2 Eligibility criteria

#### 2.2.1 Type of study

Only randomized controlled trials (RCTs) can be included. Observation studies, animal research, case report, review, and meta-analysis are excluded.

#### 2.2.2 Participants

Patients who have been diagnosed with T2D will follow the American Diabetes Guidelines. There are no restrictions on gender, age, course of the disease, TCM syndrome, and race.^[11]^ These types of patients will not be included: patients with acute complications of diabetes; patients with severe heart disease, liver and kidney dysfunction, mental illness, or a relevant drug allergic history and patients during pregnancy or lactation.

#### 2.2.3 Interventions

Analyzed interventions included Salvia miltiorrhiza used as monotherapy, Chinese herbal compound prescription and related alternative therapies.

#### 2.2.4 Comparison

Patients diagnosed with type 2 diabetes but who have not received it or who have only received nonpharmacological treatment.

#### 2.2.5 Outcome measures

The primary outcomes include

1. 2 hour plasma glucose
2. Fasting plasma glucose
3. Hemoglobin
4. Homeostasis model assessment of insulin resistance
5. Fasting plasma insulin and adverse events.

### 2.3 Information source

We search the following databases from January 2010 to July 1, 2020: China National Knowledge Infrastructure, China Biological Medicine, China Scientific Journals Database, Wanfang database, PubMed, EMBASE. We will search English and Chinese articles for review, and collect additional references from review references and original research articles.

### 2.4 Search strategy

Two review authors will search the literature independently with cross- check. Any inconsistency will be solved by a third reviewer. Manual search will be performed if relevant literatures are found in the included studies. The electronic search will be conducted using a combination of following keywords diabetes, T2D, type 2 diabetes, Non-Insulin-Dependent diabetes mellitus, Chinese patent medicine, Chinese herbal drugs, herbal, salvia miltiorrhiza, salvia, dan shen, randomized controlled trial, controlled clinical trial, trial, RCT, randomized, randomly. The search strategy for PubMed is presented in Table 1 and the strategy will be modified upon the requirement of other databases.

**Table 1:** XXX.

No	Search terms
#1	T2D
#2	type 2 diabetes
#3	diabetes
#4	Non-Insulin-Dependent Diabetes Mellitus
#5	Chinese patent medicine
#6	Chinese herbal drugs
#7	Chineseproprietary medicine
#8	#1 OR #2 OR #3 OR #4 OR #5 OR #6 OR #7
#9	salvia miltiorrhiza
#10	salvia
#11	dan shen
#12	#9 OR #10 # OR 11
#13	randomized controlled trial
#14	controlled clinical trial
#15	trial
#16	RCT
#17	randomized
#18	randomly
#19	#13 OR #14 OR #15 OR #16 OR #17 OR #18
#20	#8 AND #12 AND # 19

Search strategy for the PubMed.

### 2.5 Data collection and analysis

#### 2.5.1 Study selection

**Figure 1:**
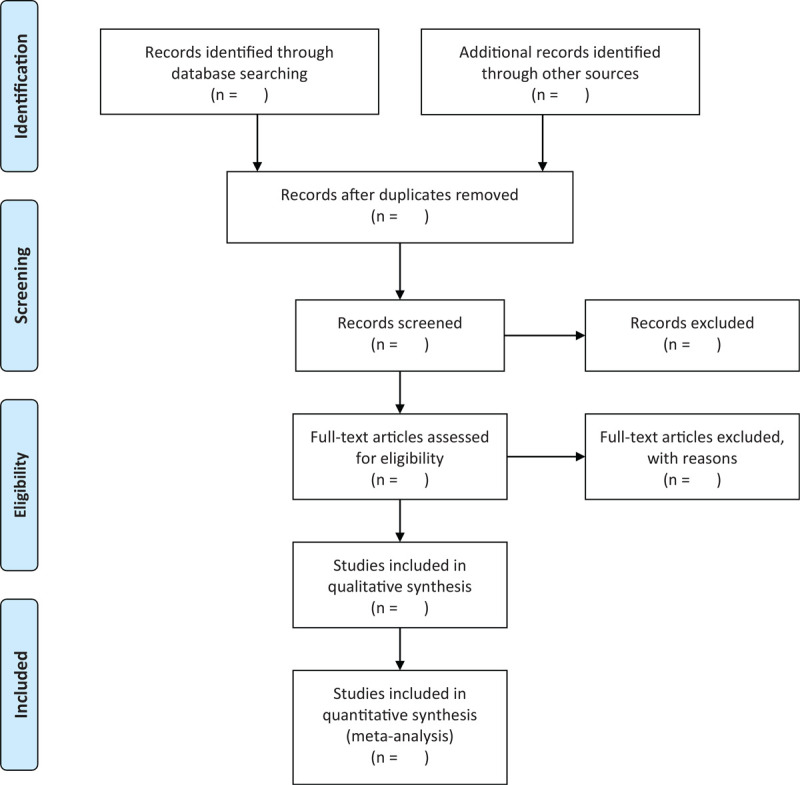
Flowchart of study selection.

Two reviewers will perform literature screening, study selection, and data extraction independently. The literature obtained will be imported into EndnoteX9 to screen the title and abstract, the duplication and studies failing to meet the pre-specified inclusion criteria will be excluded. After reading the full text of the remained literature and discussing within the group, the final included studies will be determined. The corresponding author of original RCT will be contacted when the full text is unavailable. Disagreements will be solved by consulting a third-party arbitrator or discussing within a group. The flowchart of studies searching process is shown in Figure 1.

#### 2.5.2 Data extraction and management

For each RCT, we extracted the following information:

1. general information, including name of the first author and year of publication;
2. participant characteristics, including sample size, T2D severity, gender composition, mean age, and diagnostic criteria;
3. intervention details, including the experimental medicine and control group care, doses of medications, duration of treatment; and
4. outcome measures and intergroup differences.

Extract data from the primary and secondary results for further evaluation. The inconsistency between the 2 reviewers will be resolved by the third reviewer.

#### 2.5.3 Risk of bias in included studies

The quality of the studies will be assessed by using the Cochrane Handbook 5.1.0 (Cochrane Handbook 5.1.0). The assessment will include random sequence generation, randomization correctness, allocation scheme hiding, blinding of patients and implementers, accuracy of data results, and other risk of bias. The risk of low bias is expressed as “low risk” and the risk of high bias is expressed as “high risk”. The information provided in the studies is inaccurate or does not provide sufficient information for the bias assessment to be expressed as “unclear risk”. The above content evaluation was independently evaluated by 2 researchers, and any differences will be resolved through discussions with the third reviewer.

#### 2.5.4 Measurement of treatment effect

Two reviewers will analyze the data independently using RevMan 5.3.5 Risk ratio with 95% confidence interval will be adopted for the dichotomous data, whereas the mean difference or standardized mean difference with 95% CI will be utilized for the continuous data.

## 3 Assessment

Assessment of reporting biases. A funnel plot will be performed to assess any publication bias when more than 10 RCTs are included. In additional, Egger regression and Begg correlation test will also be performed to identify the funnel plot asymmetry. The Cochrane *I*^2^ and Q tests will be applied to evaluate the heterogeneity with the cut-off value of *I*^2^ = 50. If *I*^2^ > 50% and/or Q test <0.10, the heterogeneity will be deemed significant.

## 4 Data synthesis

In line with the Cochrane guideline, a fixed-effect model will be utilized to pool and analyze the outcome data if *I*^2^ < 50, and a random-effect model will be employed if *I*^2^ ≥ 50. Subgroup analysis or meta-regression will be performed to assess the potential sources and present reasonable explanations for the heterogeneity. Sensitivity analysis will be applied to evaluate the stability of the pooled results of included RCTs according to the methodological quality, sample size and missing data.

## 5 Grading the quality of evidence

The Grading of Recommendations Assessment, Development and Evaluation (GRADE) guidelines will be utilized to grade the quality of evidence as very low, low, moderate, or high.

## 6 Discussion

Diabetes has become a major public health problem worldwide, especially in developing countries.^[12]^ On the other hand, high blood sugar often leads to a series of complications, and severe cases can even cause death.^[13,14]^ At present, our understanding of the pathogenesis of diabetes is not fully understood, and the drugs commonly used in clinical treatment of type 2 diabetes are often accompanied by some side effects, such as hypoglycemia, liver damage and gastrointestinal discomfort, etc^[15,16]^. Therefore, people urgently need some supplementary and alternative medicines to improve the treatment of diabetes.

SM was often used with other herbs in ancient China to treat various diseases in TCM clinical practice, including diabetes (consumptive thirst in TCM). With modern pharmacology in-depth research on the components and effects of SM, an increasing number of people attach importance to SM prevention and treatment of diabetes.^[17,18]^ As a natural medicine that has been used in China for many years, SM has many advantages that synthetic medicines do not have. More and more evidence shows that Danshen can lower blood sugar and improve diabetes complications.^[19–21]^

However, currently no systematic review and meta-analysis have been conducted regarding the efficacy and safety of SM for the treatment of patient with T2D. The findings of this study may yield helpful evidence for the clinicians and investigators concerned in decision-making process about the efficacy and safety of SM for patients with T2D

This study was supported by The Project of Science and Technology Department of Sichuan Province for Youth Fund (2020JDRC0115). The authors would like to thank Sanyin Zhang and Zhipeng Wu for critically reviewing the manuscript.

## Author contributions

**Data collection**: Ying Guo, Jianfeng Sun.

**Statistical analysis**: Renyan Zhang, Jianfeng Sun.

**Supervision**: SanYin Zhang, Zhipeng Wu.

**Writing – original draft**: Ying Guo, Peng Yang.

**Writing – review & editing**: Sanyin Zhang, Zhipeng Wu.

## Abbreviations

Abbreviations: T2D = type 2 diabetes, SM= salvia miltiorrhiza, 2hPG = 2-hour postprandial blood glucose (2hPG), BMI = body mass index, FBG = fasting blood glucose, RCT = randomized controlled trials.
